# Correspondence

**Published:** 1885-03

**Authors:** 


### CORRESPONDENCE.

LONDON, ENG., February 5, 1885.

TO THE EDITOR OF THE DENTAL REGISTER:

DEAR SIR:—In my last letter I just touched upon, in a general way, some of the lax methods in operative dentistry as practiced here; what is true of operative dentistry is equally true of prosthetic dentistry.

Here the general rule is in practices of any dimensions to keep a mechanical dentist, and one, or more, boys, whose services can be secured at a nominal sum; these have charge of the prosthetic branch, the operator only taking the impressions and wax-bites (and inserting cases when completed); he also fills out blanks describing the peculiarities of the case, and kind of work desired, etc. This is, of course, very nice for the automaton in the workshop; the word laboratory is not used here, and I think under the circumstances "workshop" is the most appropriate word to use.

Of course, your readers will readily understand how well nigh impossible it would be under such circumstances as these to obtain anything like artistic results from such a slovenly method of procedure. I contend that in order to produce the best results possible, both for operator and patient, a perfect understanding of the case under consideration is essentially necessary in all its details; contour of profile, facial expression, temperament, complexion, and a general appreciation of harmony that only comes of continued contact with patients, and a careful study of all that conduces to the perfect symmetry of nature's noblest production—man; of course, the latter is supposed to embrace women (no joke intended).

In the absence of any such opportunities of observation, how is the mechanical dentist to become anything but what he is? I must leave it for some one better than I am at conundrums to answer.

That some of the older dentists were good mechanics, there is ample proof. I have seen some very fine pieces of mechanism from their hands, mechanically perfect; but with as little appreciation of the artistic or anatomical relation to associate parts as could well be studied out.

Their principal forte was metal plates, some of which were fearfully and wonderfully made, what with their "bands," "wires," "stays," "vandykes," "swivels," "clasps," "springs," etc., one can fully appreciate why the cases when made were as much anxiety to the patient as the nomenclature would be to the dentist who superintended the construction of his own cases, and thereby secures for his patients the best results, by supplying the brains and mental equipoise that the average mechanie appears to be entirely innocent of.

There are a few honest exponents of prosthetic dentistry here that endeavor to do the best thing possible for their patients, but the great proportion of work as turned out would best suit the requirements of patrons of "Steam Dental Cos.," and those firms that advertise "A rattling set of teeth for $10.00."

There seems to be a tendency in all old countries to be satisfied with following in the ruts of the musty past, this truism is quite applicable to England, and painfully so of dentistry as practiced here; and one fact not very cheering is the apathetic manner in which the people submit to antedeluvian practice, while they support with generous fees the despoilers of their natural organs.

The only thing to be done, I suppose, is for the earnest few to work and wait; but the millenium of dentistry, in so far as this country is concerned, appears to be very, very distant, and ere it arrives you may hear again from

Yours, very truly, "78."

